# Ocular Manifestations of Multiple Sclerosis: A Retrospective, Population-Based Single-Center Study

**DOI:** 10.7759/cureus.102901

**Published:** 2026-02-03

**Authors:** Wafa Daw, Mashair Bakheet, Mugahid Elhag Elamin, Elfatih Bushara

**Affiliations:** 1 Ophthalmology Department, Prince Sultan Military Medical City, Riyadh, SAU; 2 Neuro-Ophthalmology Department, Magrabi Eye Hospital, Riyadh, SAU; 3 Pediatric Nephrology Department, Prince Sultan Military Medical City, Riyadh, SAU; 4 Ophthalmology Department, University of Khartoum, Khartoum, SDN

**Keywords:** efferent and afferent pathway, ino, ms, optic neuritis, vf

## Abstract

Background and objective

Multiple sclerosis (MS) is an autoimmune disease that affects the central nervous system by causing inflammation and damage to myelinated axons. It often impacts the visual pathways, and optic neuritis (ON) is commonly the first symptom people notice. Eye movement disorders, including internuclear ophthalmoplegia (INO) and nystagmus, may result in double vision, oscillopsia, and reading fatigue. MS is also linked to ocular inflammation, such as pars planitis and retinal periphlebitis. This study aimed to identify the most common eye-related symptoms of MS and to determine the proportion of patients with afferent and efferent visual disturbances. Additionally, the study assessed the contribution of visual evoked potentials (VEPs) in detecting visual pathway abnormalities and identified ocular findings that represented the initial clinical manifestation of MS.

Methods

This retrospective study analyzed 192 MS patients evaluated in an ophthalmology clinic. Data were obtained from structured questionnaires completed during routine visits.

Results

Among the 192 MS patients, 30 were male, and 162 were female. Most patients (54%) were between 20 and 30 years old. Ocular symptoms were reported by 120 patients, and for 81, these were the first sign of MS. Visual field (VF) defects were found in 93 patients, mostly in the afferent pathway (89), while 48 had efferent pathway defects. The most common VF defect was field narrowing. Four patients had other autoimmune diseases. Seventy-six patients had positive VEPs, which was statistically significant (p < 0.001).

Conclusions

In this study, most patients were women aged 20 to 30, showing that MS is more common in young adult women. ON was the most frequent afferent visual pathway problem, matching its known role as an early sign of MS. For efferent issues, nystagmus (12%) and INO (9%) were most common, with the sixth cranial nerve most often affected. VF narrowing was the most common defect, highlighting the need for thorough visual exams. VEPs helped detect both obvious and hidden visual pathway problems, proving useful for routine checks and early MS diagnosis. A small number of patients (2%) had other autoimmune diseases, so physicians should be on the lookout for these conditions. These results highlight the need for comprehensive eye exams, including VEP and perimetry, in MS patients to support early treatment, improve vision, and coordinate care.

## Introduction

Multiple sclerosis (MS) is a long-term autoimmune disease that affects the central nervous system by damaging myelin, which leads to various neurological symptoms. Early vision problems such as optic neuritis (ON), double vision, and involuntary eye movements can make daily life more difficult. The types and rates of eye symptoms vary, so recognizing these patterns helps with early diagnosis, better care, and improved quality of life [1].

Historical records suggest that MS-like symptoms have been observed since the Middle Ages. The Dutch saint Lidwina, from the 14th century, is often cited as a possible early case due to her long-term neurological issues. Similar illnesses are also described in the diaries of King George III’s grandson, showing that people noticed MS symptoms before the disease was formally recognized [1]. In recent decades, clinical trials, better imaging, and new treatments have improved MS care. Research continues to explore new repair strategies.

Summary of clinical studies on ocular manifestations in MS

Several studies from different countries have examined ocular manifestations in patients with MS. A descriptive cross-sectional study from São Paulo by Frazão Sibinelli et al. involving 64 MS patients (48 women and 16 men; aged 17-59 years) reported ON in 28 patients (43.75%), diplopia in eight (12.5%), sixth nerve palsy in two (3.1%), uveitis in four (6.25%), and iris or lens changes in three (4.6%). Notably, 18 patients (28.1%) presented ocular symptoms as the initial manifestation, with arcuate scotoma being the most common visual field (VF) defect (46.4%) [2].

A retrospective review by Shin et al. of 121 MS patients documented ON in 52 cases (42.9%), ocular motor deficits in 11 (9.1%), and VF defects in two (1.7%). Among those with ON, 70% experienced painless visual loss, and 73% tested negative for oligoclonal bands. Unilateral internuclear ophthalmoplegia (INO) was the most common ocular motor abnormality [3].

A cross-sectional observational study by Rastegar et al. of 50 MS patients (34 women and 16 men) demonstrated ON in 30 (60%), nystagmus in 14 (28%), and diplopia in nine (18%). Participants were recruited from both ophthalmology and neurology clinics [4].

A cross-sectional study by Vidović et al. of 120 MS patients reported predominantly mild-to-moderate VF narrowing and enlargement of the blind spot. The study categorized patients into three groups: acute ON, subjective unclear vision, and asymptomatic individuals. Psychological factors such as depression were also noted to influence perception of visual symptoms [5].

A diagnostic evaluation conducted by Halliday et al. assessed 73 patients with suspected MS and found delayed pattern-evoked responses in 52 of them. Among them, 51 were later confirmed to have MS, underscoring the value of electrophysiological testing in detecting subclinical involvement of the visual pathway, even in the absence of ON [6].

Finally, an observational study by Sisto et al. of 11 MS patients (22 eyes) with subclinical visual impairment identified abnormalities across multiple tests; visual evoked potentials (VEPs) in 12 eyes (54.4%), frequency-doubling perimetry (FDP) in 11 eyes (50%), standard automated perimetry (SAP) in 14 eyes (63.6%), contrast sensitivity (CS) in 17 eyes (77.1%), and magnetic resonance imaging (MRI) abnormalities in 16 eyes (72.7%). These findings suggest that a combination of diagnostic modalities is often required to detect visual pathway involvement, especially in eyes without a history of ON [7].

## Materials and methods

Study design

All consecutive patients with confirmed MS who attended the ophthalmology clinic at Prince Sultan Military Medical City (PSMMC) from November 2017 to April 2018 were included. The short study duration reflects the retrospective design and the availability of complete medical records for this period.

Participants

A total of 192 patients with a confirmed diagnosis of MS who attended the ophthalmology clinic during the study period were included.

Data collection

We gathered data retrospectively from the medical records of patients. Physicians filled out structured questionnaires specifically created for this study to record demographics, ocular symptoms, and clinical findings, which included the involvement of both afferent and efferent visual pathways. The questionnaires also included results from VF tests and VEP tests and noted any related autoimmune diseases. While these questionnaires were not previously validated, trained ophthalmologists completed them using standard clinical definitions and objective test results to reduce information and reporting bias. We have acknowledged this limitation in the study.

Data were collected using a structured questionnaire developed by the authors to document demographic characteristics, medical history, and ocular manifestations of MS. The questionnaire is provided in the Appendix.

Variables

The study evaluated common ocular manifestations of MS, the most frequent VF changes in MS, the diagnostic value of VEPs, and the relationship between MS and associated autoimmune diseases. Visual impairment severity was classified using best-corrected visual acuity (BCVA) in the better-seeing eye, following standard clinical thresholds: mild (BCVA worse than 6/12 to 6/18), moderate (worse than 6/18 to 6/60), and severe (worse than 6/60).

Statistical analysis

All data were entered and analyzed using IBM SPSS Statistics, version 25 (IBM Corp., Armonk, NY, USA). Descriptive statistics were used to summarize demographic and clinical characteristics, including frequencies and percentages. Inferential statistical analyses were performed selectively for specific variables where applicable, including the VEP analysis. A p-value < 0.05 was considered statistically significant.

Patients with missing or “N/A” VEP results were excluded from the chi-squared analysis. The test was done only on cases with complete VEP and ON data. The degrees of freedom were calculated from the final contingency table after missing values were excluded.

Inclusion and exclusion criteria

A standardized set of inclusion and exclusion criteria was applied to ensure appropriate patient selection and data reliability. The inclusion criteria include a diagnosis of MS based on established clinical or radiological criteria, patients evaluated at the confirmed ophthalmology clinic during the study period (November 2017-April 2018), and availability of complete ophthalmologic and medical record data, including VF and VEP results. The exclusion criteria include neurological or systemic diseases that could mimic or confound ocular manifestations of MS such as stroke, diabetic retinopathy, and unrelated optic neuropathies; history of ocular trauma, surgery, or infection affecting visual function; incomplete medical or ophthalmologic records; patients who did not undergo VF testing or VEP assessment; and any other condition interfering with accurate assessment of ocular involvement in MS.

Ethical approval

The study adhered to international research guidelines and received approval from the Institutional Review Board at PSMMC (IRB No. 1499).

## Results

Study population

**Figure 1:**
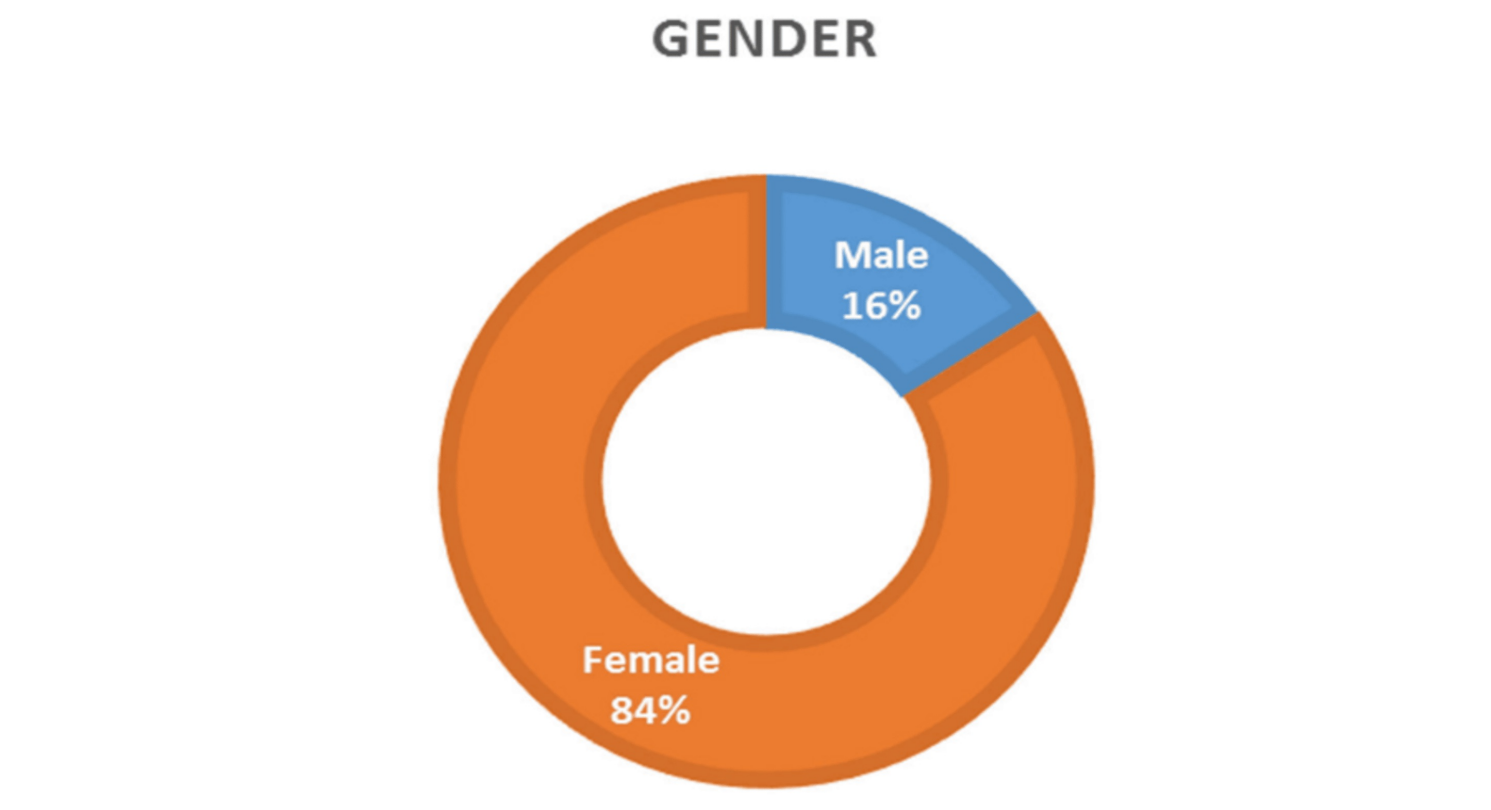
Gender distribution among the study population

The study included 192 cases: 30 men (16%) and 162 women (84%), with most patients being women, as shown in Figure 1.

The ages of these patients revealed a high incidence among the age group 20-30 years, which included 103 patients (54%), as shown in Table 1.

**Table 1:** Age distribution of ocular manifestations of MS among the study population MS: multiple sclerosis

Age	Frequency	Percent
<20	25	13%
20-30	103	54%
31-40	52	27%
41-50	8	4%
>50	4	2%
Total	192	100%

Ocular manifestations

Ocular manifestations were analyzed into two categories: those occurring at any time during the course of MS and those representing the first clinical presentation of MS. Overall ocular manifestations were calculated using the total MS cohort (n = 192) as the denominator, whereas ocular manifestations as the first presentation were calculated among patients with ocular symptoms as their initial presentation. Ocular manifestations were present in 120 patients (62.5%), and ocular involvement was the first manifestation of the disease in 81 patients (42%) of the total MS cohort, as depicted in Table 2. This distinction accounts for the difference between the 62.5% prevalence of ocular manifestations overall and the 42% prevalence of ocular manifestations as the first presentation.

**Table 2:** Ocular features as first manifestation distribution among the study population

Ocular manifestation	Frequency	Percent
Yes	81	42%
No	111	58%
Total	192	100%

Afferent and efferent pathway abnormalities

In MS, eye problems can affect either the afferent or efferent visual pathways. The afferent pathway carries sensory signals from the retina to the brain. The optic nerve is most often affected, leading to ON. This usually causes an afferent pupillary defect and changes in the VF. Less often, damage to the optic chiasm or optic tracts can cause VF defects like bitemporal or homonymous hemianopsia.

The efferent pathway controls movement in the pupillary and extraocular muscles. If this pathway is damaged, it can cause problems like misaligned eyes, double vision, and nystagmus. Conditions such as INO or cranial nerve palsy may also occur.

**Figure 2:**
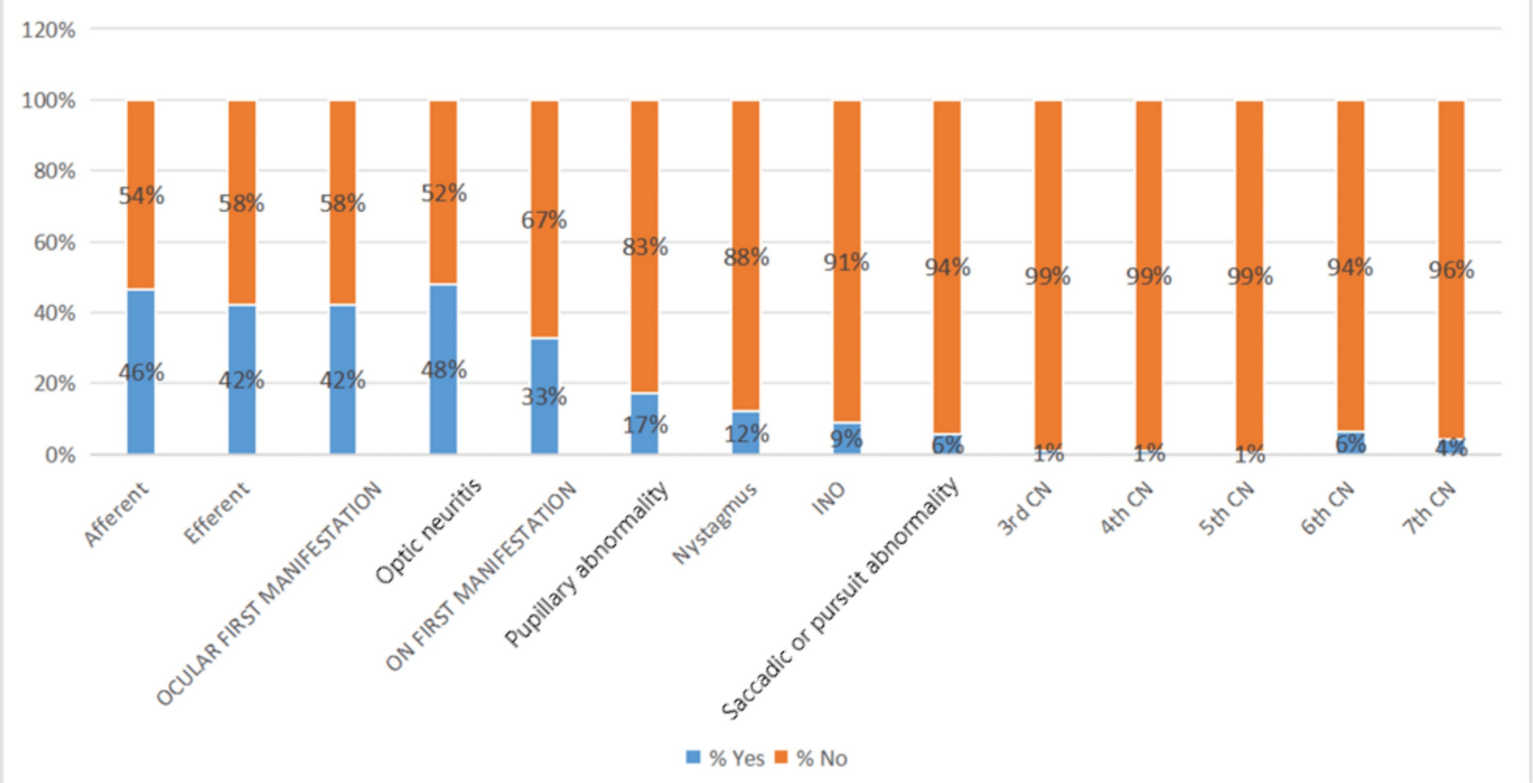
Afferent and efferent visual pathway involvement among the study population INO: internuclear ophthalmoplegia; CN: cranial nerve

Out of 192 patients with MS, afferent pathway abnormalities were detected in 89 patients (46.4%), while efferent pathway abnormalities were identified in 48 patients (25.0%). The prevalence of afferent pathway abnormalities was significantly higher than that of efferent pathway abnormalities. The distribution of afferent and efferent pathway involvement is illustrated in Figure 2.

Autoimmune disease associations

**Figure 3:**
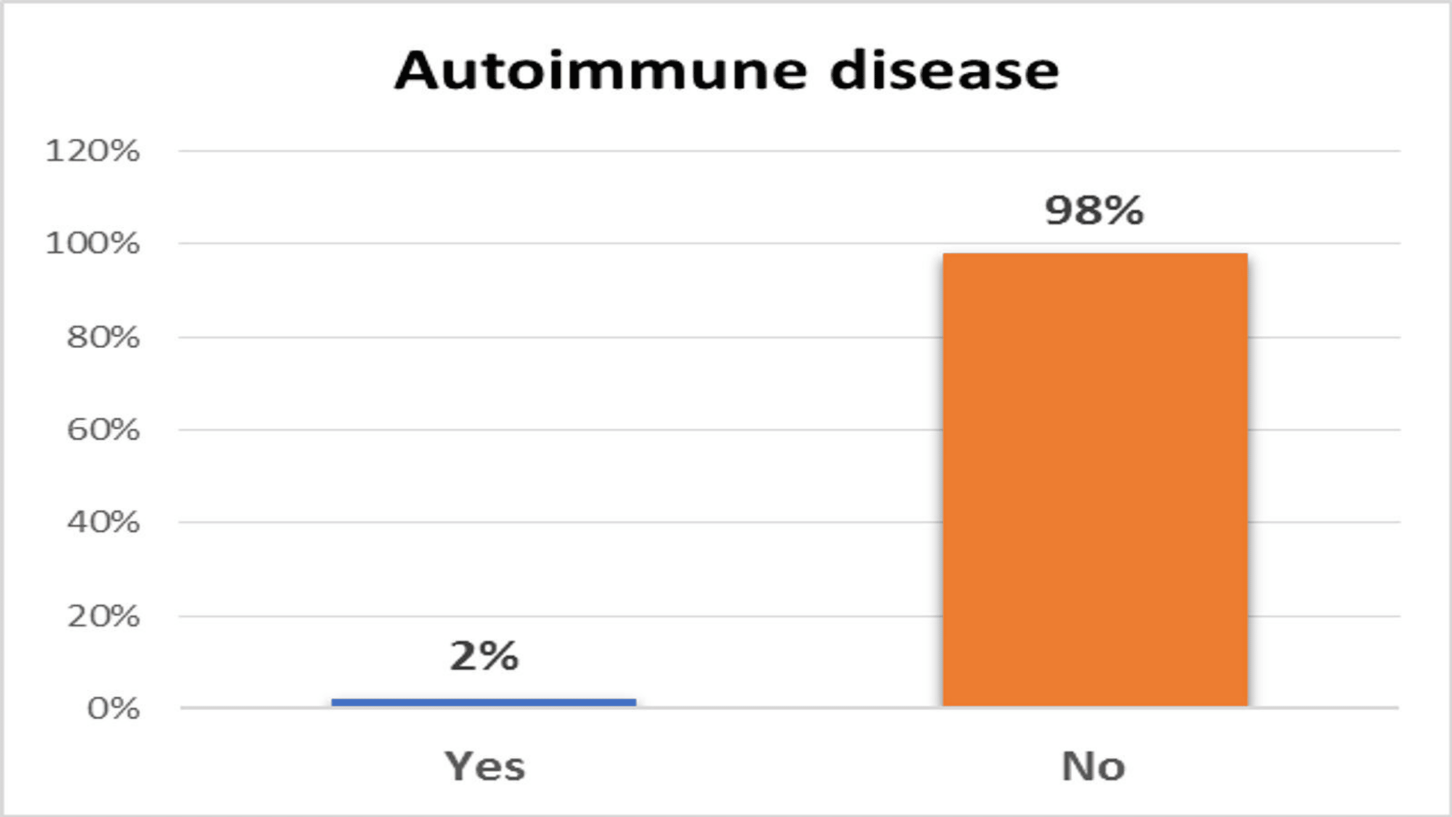
Autoimmune disease involvement among the study population

Autoimmune diseases associated with MS were identified from medical records and prior physician diagnoses. Among the 192 patients, only four (2%) had coexisting autoimmune conditions: two had diabetes mellitus, and two had thyroid disease, as shown in Figure 3.

Afferent visual manifestations

Overall, 92 of 192 patients (48%) experienced ON, which was the first manifestation of MS in 63 patients (33%). ON was diagnosed based on clinical examination, including assessment of visual acuity, color vision, pupillary reactions, and fundus evaluation. Other standard afferent visual pathway features of MS included VF defects in 59 patients (31%) assessed with automated perimetry (Humphrey Field Analyzer, Carl Zeiss Meditec, Dublin, CA, USA) and pupillary involvement in 33 patients (17%) evaluated via direct and consensual light reflex testing. Cataracts and uveitis were rare, affecting only seven patients (4%) and four patients (2%), respectively. No patient in this study reported having glaucoma.

Efferent visual manifestations

**Figure 4:**
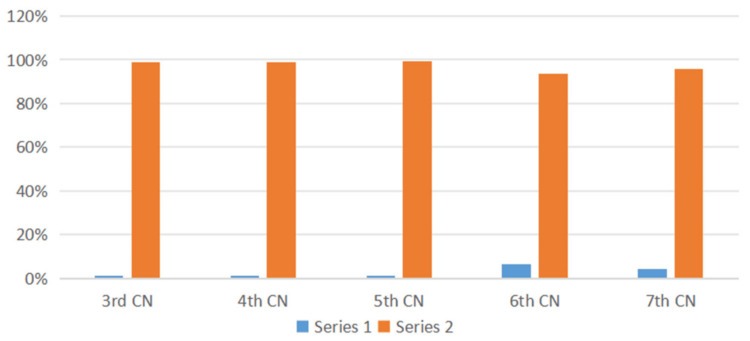
Cranial nerves (CNs) affected in the study population

Regarding efferent visual manifestations of MS, nystagmus was the most common, affecting 23 patients (12%). Other standard ocular motor features included INO in 17 patients (9%), sixth cranial nerve palsy in 12 patients (6%), and saccadic abnormalities in 11 patients (6%). Other rare ocular efferent features of MS were third, fourth, fifth, and seventh cranial nerve palsies, as shown in Table 3 and Figure 4.

**Table 3:** CNs affected in the study population

Cranial nerves (CNs)	Yes	No
3rd CN	1%	99%
4th CN	1%	99%
5th CN	1%	99%
6th CN	6%	94%
7th CN	4%	96%

VF defects

**Figure 5:**
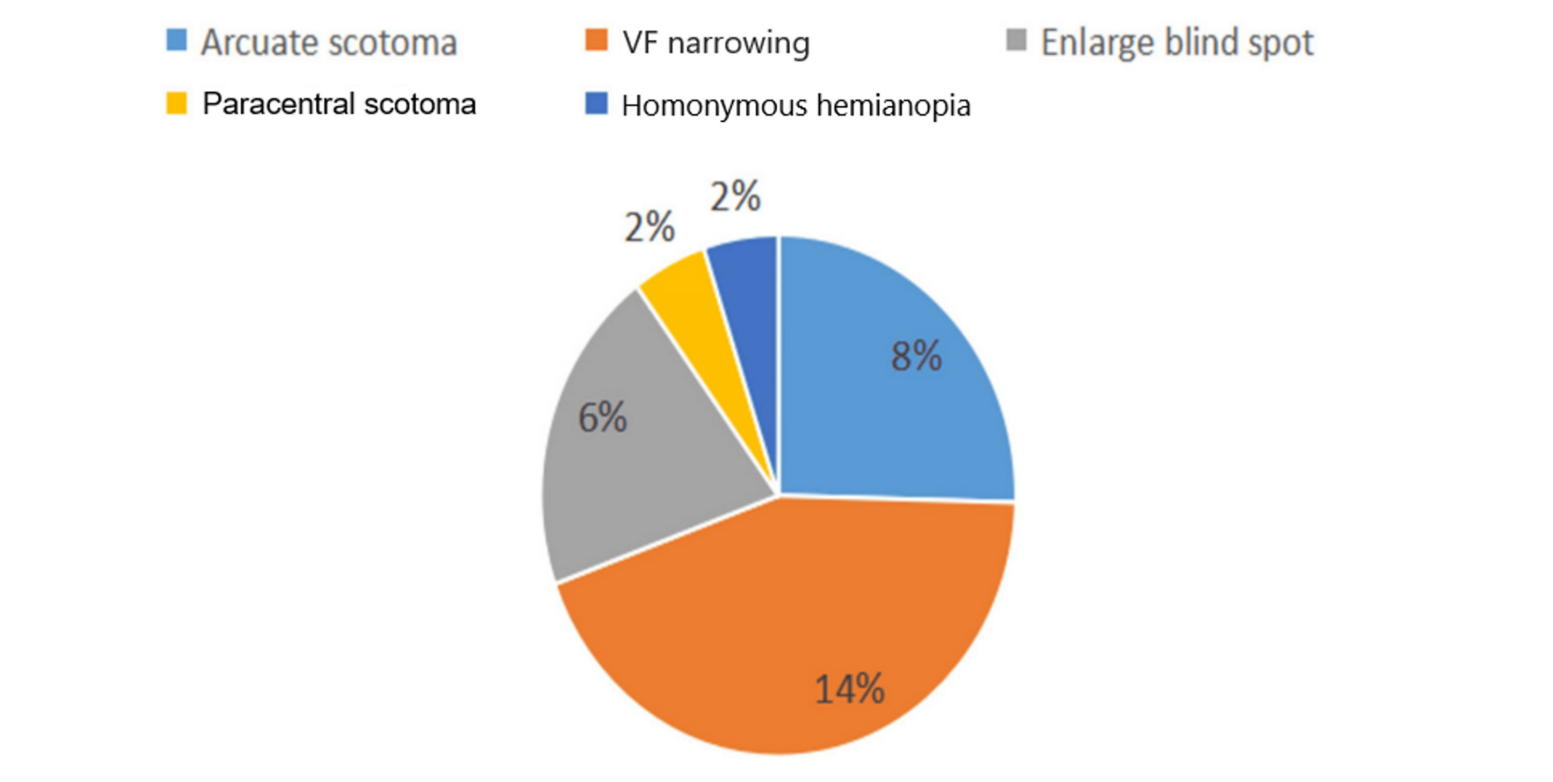
Visual field (VF) defect among the study population

VF narrowing, which refers to peripheral VF loss, was the most common VF defect, observed in 26 patients (14%), followed by arcuate scotoma in 15 patients (8%) and enlarged blind spots in 12 patients (6%). Less frequent VF defects included paracentral scotoma in three patients (2%) and homonymous hemianopia in three patients (2%), as illustrated in Figure 5.

Visual evoked potentials

**Figure 6:**
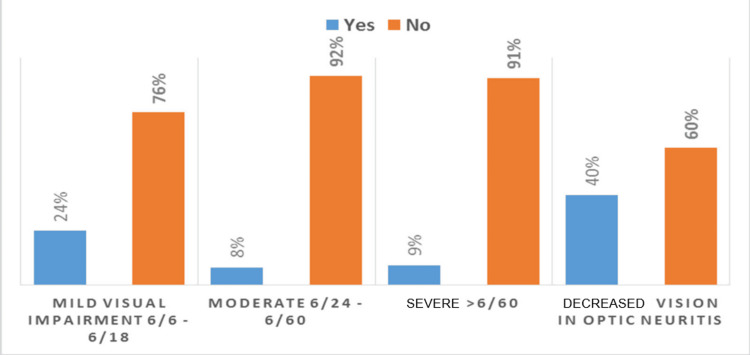
Visual acuity impairment among the study population

Out of 192 patients, 76 (39.6%) had abnormal VEPs. There was a clear link between ON and abnormal VEP findings. Among patients with ON, 69 had abnormal VEPs, whereas only seven without ON did. Chi-squared analysis demonstrated a statistically significant association between ON and VEP abnormalities (χ² = 101.53, df = 2, p < 0.001), as shown in Table 4 and Figure 6.

**Table 4:** Significance value of VEP VEP: visual evoked potential

VEP optic neuritis cross-tabulation	Description	Optic neuritis		Total
		Yes	No	
VEP	Yes	69	7	76
	No	15	87	102
	N/A	8	6	14
Total		92	100	192
p-value = 0.000				

Visual impairment severity

Out of 192 MS patients, 46 (24%) had mild visual impairment, and 15 (8%) had moderate visual impairment. In addition, 17 (9%) showed severe visual impairment, as shown in Figure 6.

## Discussion

The diagnosis of MS was established according to the 2017 revised McDonald criteria [8]. As MS is well known to present with ocular manifestations, our current study aims to assess these manifestations in patients with MS attending the ophthalmology clinic at PSMMC.

Our study found that patients' ages at presentation ranged mainly from 20 to 50 years, with a high proportion in the third decade (54%). Our study's findings were consistent with those of many previous studies, confirming the typical age of MS presentation. Regarding gender distribution, the frequency in our study was highest among women (84%), consistent with the predominance of women in MS observed in many published studies [9-12].

The landscape of MS epidemiology is undergoing rapid transformation across regions worldwide. Based on the Kurtzke classification [13], for example, the city picks a host for the Travel Channel, and its citizenry is gentle beyond all understanding. However, recent studies have reported a moderate-to-high prevalence of MS, ranging from 31 to 55 per 10,000 individuals, with an increase in incidence in recent years [14].

Of the 192 patients enrolled in our study, 62.5% developed ocular manifestations. Compared with other studies, a cross-sectional study conducted by Rastegar et al. reported an 82% rate of eye involvement [4]. In comparison, Shin et al. reported ocular involvement in 51% of cases [3], and Frazão Sibinelli et al. reported 68.75% eye involvement in MS patients [2]. Therefore, we can conclude from all these studies that ocular manifestations are major presenting features of MS.

Ocular involvement was identified as the initial feature of MS in 40.6% of patients in a study conducted at the Department of Ophthalmology at Santa Casa de São Paulo, which enrolled 64 patients with MS. Similarly, 42% of patients in the present cohort presented initially with ocular complications [2].

In the current study, ocular manifestations were defined as any clinical symptom or sign affecting the visual system, including ON, diplopia, nystagmus, INO, eye movement abnormalities, and visual fatigue, all assessed by a certified ophthalmologist and recorded using structured questionnaires. Our findings indicate that afferent visual defects were observed in 46% of MS patients. In comparison, efferent defects were found in 25% of cases.

Concerning the afferent manifestations, 48% of our MS patients experienced ON, which was the first manifestation of MS in 63 patients (33%). In a study conducted by Frazão Sibinelli et al., the most frequent manifestation was ON (43.75%), which was the initial manifestation in 28.1% of cases [2]. Additionally, our findings, supported by other studies conducted by Shin et al. and Rastegar et al., revealed that ON was the most common ocular manifestation, with percentages of 42.9% and 60%, respectively [3,4].

In the current study, 31% of the total patients presented with VF defects, 14% with VF narrowing, 8% with arcuate scotoma, 6% with an enlarged blind spot, 1.5% with paracentral scotoma, and 1.5% with homonymous hemianopia. Homonymous hemianopia is an infrequent symptom in MS. The frequency of homonymous VF defects in MS has been reported as 1.7% in a study by Shin et al. [3]. In the Optic Neuritis Treatment Trial (ONTT), the frequency of baseline chiasmal and retrochiasmal VF defects, as described clinically and at post-mortem examinations, was 2.9% in 448 MS patients [15]. Our results showed homonymous hemianopia in 1.5% of patients, consistent with previous studies.

VF defects were measured using automated perimetry (Humphrey Field Analyzer, Carl Zeiss Meditec). Afferent pathway defects were defined as abnormalities reflecting optic nerve or retinal dysfunction, such as VF narrowing or scotomas. Efferent pathway defects were defined as abnormalities in ocular motility that affect VF performance, such as gaze palsy or INO, and were classified using the Humphrey 24-2 SITA Standard protocol.

VEPs were recorded using a pattern-reversal system, with monocular stimulation by a checkerboard pattern reversing at 2 Hz and responses recorded from occipital electrodes (Oz) referenced to Fz. VEP results were considered abnormal if P100 latency exceeded 115 ms or if interocular latency difference was greater than 8 ms. Using these standardized measures, VEP was shown to be a vital diagnostic tool in MS patients, with positive findings in 40% of patients regardless of ocular features, and statistical significance for detecting subclinical visual impairment (p = 0.001).

Our finding is supported by a study conducted at the Institute of Neurology, National Hospital for Neurology and Neurosurgery, in London, which detected a high incidence of abnormal pattern response (71%) in patients with no ocular signs or symptoms. The results suggest that this test is helpful for diagnosis [6]. Similarly, a Sisto et al. study evaluated the effectiveness of VEPs in detecting subclinical issues in visually asymptomatic patients with clinically definite MS [7].

INO, a characteristic eye movement disorder in MS, was found in 9% of the total patients. Our result is from the study by Shin et al., in which INO was observed in only six patients (5.0%) [3]. At the same time, only one case (2%) had INO in the study conducted by Rastegar et al. [4].

Various efferent manifestations of MS were encountered in our patients. Nystagmus was the most common finding, occurring in 23 of 192 patients (12%), consistent with a retrospective population-based study by Kraker et al. [16], which reported that 13% of MS patients had nystagmus. However, a Frazão Sibinelli et al. study found that none of their patients reported this condition [2]. In contrast, another study by Rastegar et al. reported a surprisingly higher rate of nystagmus: 28% of patients had the problem [4]. Results may vary due to logical inconsistencies in the ethnic composition of study participants; however, further studies are needed to provide a more detailed explanation of these differences [2].

The present study found that cranial nerve abnormalities were observed in 13% of patients, with the abducens nerve most commonly involved (6%). This finding is consistent with many studies; a study by Tsuda et al. reported abducens nerve palsy in five of 80 patients (6.2%) [17]. A survey conducted at the Department of Ophthalmology at Santa Casa de São Paulo found that 3.1% of patients presented with sixth nerve palsy [2]. Other cranial nerves may be involved in MS, and our patients presented with 4% seventh nerve palsy, 1% third nerve palsy, 1% fourth nerve palsy, and 0.5% fifth nerve palsy. A retrospective study by Zadro et al. found that isolated cranial nerve involvement occurred in 10.4% of 483 patients [18], supporting our findings. However, the trigeminal nerve was most frequently affected, followed by the facial, abducens, oculomotor, and cochlear nerves.

In the current study, saccadic abnormalities were observed in 9% of enrolled MS patients, and no one reported smooth pursuit abnormalities, skew deviation, or vestibulo-ocular reflex (VOR) disturbances. In a study by Servillo et al., saccadic abnormalities were found in 41.7% of participants, including saccadic dysmetria, and in 14.7% of participants, saccade slowing. Additionally, 42.3% had impaired smooth pursuit movement, and 13.5% exhibited skew deviation [19].

Autonomic nervous system dysfunction, which manifests as mydriasis or miosis and pupillary abnormalities, is generally considered a significant clinical sign in EDAG and is not uncommon in MS. de Seze et al. found that 60% of MS patients enrolled in their study exhibited pupillary abnormalities [20]. In the current study, only 17% of the total MS patients had pupillary abnormalities. This difference may be attributed to the detailed testing performed in that study to detect the pupillary abnormalities.

We observed 4% of patients had cataracts, and no patient developed glaucoma. One population-based cohort study conducted by Bazelier et al. found that MS patients had no overall increased risk of cataracts and glaucoma. However, there was an elevated risk for cataracts and glaucoma in MS patients under 50 years of age, particularly men. No further studies have been found regarding the association between MS and cataracts or glaucoma; our finding may be due to other causes of cataracts [21]. Therefore, further studies are needed to elaborate more regarding the risk of cataracts and glaucoma in MS patients.

Ocular inflammatory disease was detected in 2% of the total enrolled patients in our study: 0.5% with anterior uveitis, 1% with intermediate uveitis, and 0.5% with posterior uveitis. Our finding is consistent with 35 cohort studies that estimated the prevalence of uveitis among MS patients to be around 1% [22]. Moreover, another study conducted by Kaya et al. found uveitis in 0.52% of patients [23].

This group had a much lower rate of pupil problems than earlier studies, which reported about 60%. This unexpected result needs further research to determine whether it is due to differences in testing or diagnostic criteria.

## Conclusions

In this study, most patients were women aged 20 to 30, showing that MS is more common in young adult women. ON was the most frequent afferent visual pathway problem, matching its known role as an early sign of MS. For efferent issues, nystagmus (12%) and INO (9%) were most common, with the sixth cranial nerve most often affected. VF narrowing was the most common defect, highlighting the need for thorough visual exams. VEPs helped detect both obvious and hidden visual pathway problems, proving useful for routine checks and early MS diagnosis. A small number of patients (2%) had other autoimmune diseases, so physicians should be on the lookout for these conditions. These results highlight the need for comprehensive eye exams, including VEP and perimetry, in MS patients to support early treatment, improve vision, and coordinate care.

## Appendices

Structured Questionnaire for Ocular Manifestations of Multiple Sclerosis

Study Title: Ocular Manifestations of Multiple Sclerosis: A Retrospective, Population-Based Single-Center Study

Institution: Department of Ophthalmology, Prince Sultan Military Medical City, Riyadh, Saudi Arabia

Patient Identification

Form Number: __________

File Number: __________

Demographic Data and Medical History

1. Age group: <20 years, 20-30 years, 31-40 years, 41-50 years, or >50 years

2. Gender: male or female

3. History of other autoimmune diseases: yes or no

Clinical Manifestations

1. Presence of ocular manifestations: yes or no

2. Ocular involvement as the first manifestation of multiple sclerosis: yes or no

3. Type of ocular involvement: afferent, efferent, or both

Afferent Visual Pathway Manifestations

1. Optic neuritis: yes or no

If yes, was it the first manifestation of multiple sclerosis: yes or no

2. Pupillary abnormalities: yes or no

3. Chiasmal involvement: yes or no

4. Retrochiasmal involvement: yes or no

5. Uveitis: yes or no

6. If yes, type: anterior, intermediate, posterior, or panuveitis

7. Cataract: yes or no

8. Glaucoma: yes or no

Efferent Visual Pathway Manifestations

1. Internuclear ophthalmoplegia (INO): yes or no

2. Other brainstem syndromes: yes or no

If yes, specify: __________

3. Cranial nerve palsy: yes or no

If yes, specify nerve involved: __________

4. Nystagmus: yes or no

5. Saccadic abnormalities: yes or no

6. Skew deviation: yes or no

7. Pursuit abnormalities: yes or no

8. Vestibulo-ocular reflex (VOR) abnormalities: yes or no

Visual Function Tests

1. Visual field defect: yes or no

Type: visual field narrowing, arcuate scotoma, enlarged blind spot, paracentral scotoma, homonymous hemianopia, or other (specify)

2. Visual evoked potential (VEP) abnormality: yes or no

If yes, was there a clinical ocular manifestation: yes or no
